# Inhaling Democracy: Cigarette Advertising and Health Education in Post-war West Germany, 1950s–1975

**DOI:** 10.1093/shm/hkv004

**Published:** 2015-03-15

**Authors:** Rosemary Elliot

**Keywords:** smoking, tobacco industry, West Germany, advertising health education, youth culture, social market economy, neoliberalism

## Abstract

In the late 1960s and early 1970s, the West German government was faced with the challenge of addressing a damaging health behaviour, smoking, in the context of an emerging late modern democracy, when the precedent for addressing that behaviour was set in the Nazi past. This paper details the two-pronged approach which the government took: seeking restrictions on cigarette advertising, whilst educating young people to adopt positive health behaviours in the face of pressure to smoke. This approach can be understood in the social and economic context of the time: an economic commitment to the social market economy worked against restrictions on the sale of cigarettes; whilst concerns about past authoritarian structures prompted the health authorities to seek novel ways of addressing smoking, emphasising choice. In a nuanced way, post-war anti-smoking strategies were a response to West Germany's National Socialist past, but more importantly, a signal of an increasingly international outlook.

In 1974, a long article in the annual journal for young people, *Durch die Weite Welt* (‘Through the wide world’), highlighted fears about drug use in West Germany, fears amplified by ‘frantic’ public discussion about ‘fashion drugs’ such as cannabis, LSD and heroin which had grown in popularity since the late 1960s. However, the article was not illustrated by a picture of hippies or a heroin addict, but by an image of a young boy, not even out of short pants, having a cigarette placed in his mouth by long haired youths.^1^ The scene took place against the background of a Peter Stuyvesant advert, signifying the visual and literal presence of smoking in post-war West German society, and embodying the moral fears surrounding smoking among young people. Despite coverage accorded to illicit drug use, the author of the article, Dr Feser from the *Bundeszentrale für Gesundheitliche Aufklärung* (the Federal Centre for Health, hereafter BZGA), argued that increasing youth consumption of ‘every day’ drugs, such as tobacco and alcohol, was a far greater concern than illicit drugs.^2^

The article discussed the effect of cigarette advertising, suggesting that advertising created particular expectations in the minds of consumers, from pleasant physical effects to the promotion of a lifestyle linking smoking with social success, acting to encourage smoking.^3^ The message of the image and text was clear: children were at risk not only from the habits of the ‘beat’ generation but from consumer culture and unfettered cigarette advertising. Given the danger of smoking to health, the author argued it was necessary to counter such advertising with health education—the goal of which should not be to create ‘anti’ advertising, but to make clear to children and young people the errors of adult ways. Once children were aware of the errors of adult behaviour, they could then ‘come up with good reasons to follow the new trend and not smoke’. The author explained the BZGA had been running a health education campaign under the slogan ‘*Der neue Trend*—no smoking please!’^4^

The *Neue Trend* campaign was heralded as a central plank of West German tobacco policy in the late 1960s and 1970s. The campaign, aimed primarily at young people, is significant firstly because it epitomises a contradiction evident in West Germany's tobacco policy during the second half of the twentieth century: recognition of the dangers of smoking coupled with a widespread acceptance of tobacco use among the adult population. This contradiction is apparent in other Western countries such as the USA and the UK, where the dangers of smoking were only slowly acted upon. Proctor has argued that the tobacco industry in the United States promoted a culture of ‘denialism’ of the health risks of smoking.^5^ Brandt suggests the tobacco industry constructed controversy to delay anti-smoking legislation.^6^ In the British case, Berridge argues the slow reaction was only partly ‘denial and delay’, giving consideration to the changing context of public health, the cooperative relationship between the tobacco industry and government in the 1950s and the lack of a significant anti-smoking lobby until the early 1970s.^7^ In the German case, this paper argues there was an acceptance of the health risks of smoking within the cigarette industry; indeed the issue had been acknowledged since the Nazi period. While accepting that health risks of smoking did exist, the West German cigarette industry sought to minimise government reaction to, and public perception of, those risks.

Secondly, the *Neue Trend* campaign is significant because of the content and direction of the campaign. As Berridge and Loughlin have shown, when the British government began to act on smoking in the early 1970s, through the recently formed Health Education Council (HEC), they utilised mass media and poster campaigns. These campaigns relied on graphic imagery which aimed to shock smokers and create a social stigma around smoking.^8^ The West German government explicitly rejected such an approach from the late-1960s onwards, specifically the kind of ‘anti’ advertising Dr Feser spoke against in the article discussed in the opening of this paper.^9^

Finally, this paper will reconsider the accepted idea that the West German tobacco history case is unique as a result of the experience and legacy of the Third Reich, which, scholars have argued, created a liberal post-war response to smoking.^10^ Historiography on smoking in post-war Germany has developed from what was almost a passing statement by Robert Proctor in 1999; namely that ‘popular memory of Nazi tobacco temperance may well have handicapped the post-war German anti-tobacco movement’.^11^ Scholarship from a public health perspective suggested that liberal attitudes combined with tobacco industry influence in Germany hindered European and global anti-smoking initiatives in the early 2000s.^12^

The perception of a liberal stance on tobacco use in post-war West Germany is justified by the fact that the only substantial piece of legislation on smoking prior to the early 2000s was a clause in the 1974 *Lebensmittelgesetz*, or Food Law, a consumer protection law, which prohibited TV and radio advertising of cigarettes.^13^ This law replaced a voluntary agreement to restrict cigarette advertising to young people negotiated between the West German government and the cigarette industry in 1965–6 (extended in 1971).^14^ According to Cooper and Kurzer, ‘Nazi efforts [against smoking] seem to have created an unspoken backlash against the puritanical qualities of their health campaigns, resulting in post-war resistance or inhibitions towards anti-tobacco campaigns by the government or medical profession'.^15^ However, the *Neue Trend* campaign shows that addressing smoking remained on the agenda.

Grüning, Strünck and Gilmore have argued that the impact of the National Socialist regime on post-war smoking policy was indirect: namely, the invasive nature of Nazi public health policy led to health being viewed as a private matter in the post-war period, and to a ‘dearth’ of research into public health and epidemiology within Germany.^16^ As the lead in tobacco research was taken by Great Britain and the United States from 1950 onwards, the effect of German withdrawal from the research field was, they argue, compounded by the fact that Germany's health policy was formed within a national framework, rather than in the context of international developments. Grüning and colleagues suggest that ‘a large non-English speaking country with self-sufficient health research and policy-making capacities could be entirely inward-looking and isolate itself from scientific and “policy learning” from abroad’, further contending that German language textbooks and medical training impeded the introduction of new scientific evidence on tobacco-related issues ‘from the English-speaking medical community elsewhere’.^17^

While there is a long tradition of health being viewed as a private matter in Germany, which was interrupted by the Nazis, and it is clear that the locus of smoking research shifted to Britain and North America in the post-war years, the argument that West Germany was ‘inward-looking and isolated’ is harder to sustain.^18^ West German tobacco policy was not framed in ignorance of international evidence, but in full knowledge and cognisance of it. Archival documents suggest an awareness of what was going on in other countries and, in the case of health education, West Germany's contribution to those debates. This was apparent in discussion in the Federal Ministry of Health (*Bundesministerium für Gesundheitswesen*, hereafter BMG) in the mid-1960s about the effectiveness of British health education campaigns, the lengthy English-language bibliographies in BZGA-commissioned reports, and in West Germany's presence at international conferences on smoking. It was also apparent in the discussion and debates around potential restrictions on television and radio advertising of cigarettes. Both sides of the debate (government health bodies and the cigarette industry, mediated by the Federal Ministry of Economic Affairs [Bundesministerium für Wirtschaft]) drew on examples of policy elsewhere to make their case. The transnational context explains some similarities in policy focus and arguments in different countries on the use of health education and restrictions on cigarette advertising. However, the distinct social and economic context of post-war Germany explains both the emphasis on democracy and citizenship in health education; and the delayed introduction of any meaningful restrictions on smoking. It is here that Proctor's passing reference to the post-war liberal climate is relevant.

Nonetheless, the 1966 voluntary agreement stopped far short of the more restrictive action and legislation which medical organisations and anti-smoking campaigners within West Germany advocated, both then and in the run up to the passage of the Food Law prohibiting TV and radio advertising in 1974.^19^ A comprehensive programme, proposed in the *Bundesrat* (the legislative body representing the federal states, or *Länder*), to protect non-smokers from second-hand smoke was subsequently watered down and the issue remitted to health protection agencies and the federal states.^20^ Bornhäuser, McCathy and Glantz argue that this policy dilution was because key German scientists looking at tobacco related questions were funded by the tobacco industry from the 1970s onwards, undermining independent research development and shaping policy decisions.^21^ From the 1980s onwards, according to Grüning *et al.*, the tobacco industry successfully emphasised the economic contribution of tax revenue and the importance of employment provided by tobacco manufacture and sales to the economy to counter discussion of smoking restrictions.^22^

While these points were undoubtedly the case; the economic contribution of tobacco was a key plank of the cigarette industry's lobbying strategy from the early 1950s, if not before.^23^ This paper will argue that, whilst the BMG was aware of international epidemiological evidence on smoking, the Ministry was constrained in its response by established domestic relationships and networks between the cigarette industry, the scientific community and other branches of government which pre-dated its formation in 1961 and date back to the Third Reich. These relationships altered slightly, but not seismically, with the foundation of the BZGA. Further, while such networks existed in other countries, the economic issues at stake in the German case stretched to the very conceptualisation of West German reconstruction.^24^ The economic circumstances of post-war West Germany, that is, the commitment to free trade within the context of the social market economy, worked against introducing restrictive practices, seen as ‘economic authoritarianism’, in the sale, marketing and advertising of cigarettes (and other goods). This is apparent in the difficulties that arose in getting even a voluntary agreement on tobacco advertising accepted within the post-war West German liberal economic context. The commitment to free trade within the social market economy developed from war-time arguments of the Freiburg School of Economics but was also seen by the United States as central to West Germany's rehabilitation in the West.

Further, the *Neue Trend* campaign goes far beyond publicising the health risks of smoking to address what it meant to be a citizen in a modern, liberal, consumer society. This was a response to the social and political context of 1960s West Germany. Questions of liberty and democracy came to the fore with vocal protest movements against the perceived authoritarianism of the previous decades, and generational tensions around the issue of the National Socialist past, emerging youth culture and the international climate of the Cold War.^25^ The pressure for democracy in the context of liberal citizenship raised issues for the West German government over how to shape individual behaviour without being repressive, when the precedent for government intervention on smoking was the National Socialist period, and when smoking was seen as routine and socially acceptable.

The health education campaign links to the context of economic liberalism: by the early 1970s, smoking policy was clearly informed by the liberal ideal of a citizen exercising choice, informed by experts, in a market economy. Thus, the *Neue Trend* campaign can be read as an example of neo-liberal governmentality. The campaign sought to provide young people with the means of interpreting and making health choices within a largely pro-smoking culture (construed as a nexus of tobacco industry marketing, advantageous fiscal policy and familial, peer and social pressure to smoke). There was no suggestion that health education could change this culture; the aim was to teach young people to negotiate it.

Government ministers, even from the BMG, were clear that their goal in smoking policy generally, to quote a 1974 statement, was ‘not to force healthy behaviour by laws or pressure [but] to awaken reason and criticism’.^26^ The post-war West German case was different from the ‘denial and delay’ posited elsewhere: the health risks of smoking were acknowledged, but minimised by the tobacco industry, and located within the realm of individual choice by both the tobacco industry and the BMG. The debate, within the context of neo-liberal economic freedom, was around the point at which the state could intervene. As such, arguments in West Germany are relevant to understanding UK and American debates on smoking and health, and to broader arguments around state intervention in a range of health behaviours today.

## Cigarette Advertising and Health in 1950s West Germany

The risks of smoking were generally established internationally with the publication of the report by the Royal College of Physicians of London (hereafter RCP) in 1962, *Smoking and Health*, and the US Surgeon General's report, *Smoking and Health*, in 1964.^27^ The reports were covered in the news and periodical press in West Germany and discussed in parliament.^28^ This was just one of a series of flashpoints for the West German cigarette industry, rather than a pivotal moment. The industry had responded vociferously and repeatedly to Nazi anti-smoking campaigns, in order to protect their interests.^29^ This is different to the British and US scenarios where smoking had been positively endorsed through the Second World War.^30^ In the post-war years, tobacco firms and trade organisations lobbied the new West German government in support of the re-establishment of the domestic tobacco industry in the face of the smuggling of Virginia cigarettes and international competition.^31^ This lobbying positioned tobacco as an important element of post-war economic and trade policy, despite lingering concerns about the health risks of smoking in the early 1950s. Thus, the West German cigarette industry had substantial experience of lobbying the government in the face of health and other concerns.

Through the 1950s, the West German cigarette industry responded to emerging international evidence on smoking and health by engaging prominent scientists, such as Druckerey in Freiburg and Wynder at the Sloan Kettering Institute in New York, to work on the question of smoking and lung cancer, including biological experiments to isolate the carcinogenic compounds in tobacco condensate.^32^ The West German cigarette industry set up the Scientific Research Institute of the Association of Cigarette Manufacturers (*Wissenschäftliche Forschungstelle of the Verband der Cigaretten-Industrie*; hereafter the Scientific Research Institute) in 1959 to look at both the statistical basis of risk and the toxicology of smoking, with the input of universities and the Federal Health Office (*Bundesgesundheitsamt*) itself.^33^ The Federal Health Office was the body responsible for recognising and assessing health risks in West Germany. West German cigarette manufacturers were thus able to argue they were addressing health concerns by undertaking research work to create a less harmful cigarette—with scientific and government support.

Similarly, cigarette manufacturers engaged with concerns about cigarette advertising in the West German context. Manufacturers remained governed by legislation, the *Nikotinverordnung*, implemented during the National Socialist period, which prohibited health claims about cigarettes.^34^ In the Third Reich, this related to low nicotine cigarettes, but by the 1950s, concern focused on advertising for the US Virginia blend filter cigarettes which were growing in popularity in West Germany. In 1952, Phillip Reemtsma, head of the firm Reemtsma, criticised an advertising campaign for the US filter cigarette *Gloria* (British American Tobacco) as irresponsible, believing that the slogan ‘*Genuss ohne Reue* (Enjoyment without regrets)’ misled consumers into believing they could smoke without harm.^35^ West German manufacturers were uneasy about the shift in the market towards US Virginia blend filter cigarettes; arguably, it suited them to adopt a strict position on advertising which suggested the health benefits of filter cigarettes.^36^

Reemtsma's comments also reflect concern that overstepping the line regarding health claims for cigarettes would incite opposition from ‘the medical enemies of the cigarette’ and lead to statutory regulation through the Food Law.^37^ The German Agency against Addiction (*Deutsche Hauptstelle gegen die Suchtgefahren*, hereafter DHS) registered its alarm at the proliferation and content of cigarette advertising in 1957, citing the increase in ‘suggestive’ advertising and the need to protect the family, in particular children, from the dangers of alcohol and tobacco use. This put the issue within a moral as well as a medical framework.^38^

Correspondence about the 1966 voluntary agreement from the *Verband der Cigarettenindustrie* (the trade association representing West German cigarette manufacturers; hereafter VdC) explains that the VdC had been careful since the mid-1950s not to participate in ‘Americanised’ advertising which emphasised health arguments for filter cigarettes.^39^ Correspondence in the Reemtsma archive dating from 1959 refers to an earlier agreement between member firms and the Federal Health Office (*Bundesgesundheitsamt*) not to advertise ‘particular effects’ of filters.^40^ This agreement allowed the industry to argue they were restricting health claims about filters in advertising, and persuade the Federal Ministry of the Interior, responsible for public health legislation at that point, that a re-drafting of the *Nikotinverordnung* was unnecessary.^41^ Thus, many of the arguments which came to the fore in the 1960s around smoking, cigarette advertising and health had their origins in the previous decade.

## Restricting Advertising and the 1957 Anti-Cartel Law

By the time of the publication of the RCP report in 1962, there were established relationships between the cigarette industry, scientists and sections of the West German government which helped shape the policy response in ensuing decades. The structure of the West German health system meant that policy on smoking and health had been largely devolved to the *Länder* since 1949.^42^ There was no national body, prior to the founding of the BMG in 1961, to address concerns about smoking. The BMG was created from different parts of existing government ministries, which explains the persistence of existing networks.^43^

Health Minister, Elisabeth Schwarzhaupt, was interested in smoking from the outset. Following publication of the RCP report, Schwarzhaupt asked the Commission on Tobacco and Health, set up under the auspices of Federal Health Office at her request, to review the evidence in the report.^44^ The recommendations of the Commission guided policy to come. The Commission accepted RCP report findings that smoking was injurious to health; with the caveat that statistical associations, on which much of the evidence was based, were not enough to prove causation. Whereas the RCP report concluded that animal experiments were of no relevance to proving the cause of lung cancer, the West German Commission believed such experiments were valuable, not least because the West German cigarette industry had funded such research for almost a decade. The Commission recommended intensifying existing research at the Scientific Research Institute looking at the statistical basis of risk and the toxicology of smoking, with the input of universities and the Federal Health Office itself.^45^ This was a continuation of arrangements which had grown up in the 1950s: the Commission dismissed the idea of setting up a centrally funded research institute into smoking and health, because of the financial loss for existing centres and the professional implications for scientists removed from their existing institutional settings.^46^ The National Socialist precedent for a centrally funded tobacco research institute was not mentioned.

The Commission made a number of broader recommendations, including that the government reach an agreement with the cigarette industry about advertising; that smoking on public transport be prohibited; that sales to young people be prohibited; that filters reach minimum requirements in terms of removing nicotine and tar products; and that health education be targeted at young people. These recommendations were similar to those put forward in the original report, although the West German committee argued the British experience showed general health education campaigns to be largely ineffective, and efforts should focus on preventing youth smoking.^47^ This fits the context of a historical precedent for targetting young people in health education dating from the First World War.^48^

Medical and anti-smoking advocacy groups within Germany were explicit in their demands for a strong response to the RCP report. An article in a weekly medical journal, the *Münchener Medizinische Wochenschrift*, entitled ‘Are the English ahead of us in research, recognition and preventive work against the dangers of smoking?’ (‘*Sind die Engländer uns voraus in Forschung, Erkenntnis und Abwehr der Gefahren des Rauchens?*’), criticised the BMG for their lack of action, citing presumed fears of losing revenue from tobacco taxes. The article discussed British research, but made no mention of research and anti-smoking propaganda during the Nazi period.^49^ The article was based on a paper at a symposium for West German doctors on smoking and health, in May 1962. The delegates put forward 12 recommendations, including prohibiting tobacco as much cigarette advertising was aimed at teenagers and young people in their twenties.^50^ Similar demands were made by the *Deutsche Hauptstelle gegen die Suchtgefahren*.^51^

Nonetheless, Health Minister Schwarzhaupt was clear that an outright ban on advertising and broader restrictions on smoking were not her aims. She stated, in response to the recommendations of the DHS in particular for a ban on advertising, in October 1962:

[T]he suggestions of the German Agency against Addiction deserve a thorough examination. I think, however, that prohibition and punishment are less likely to prevent the misuse of *Genuβmitteln* than a broadly based health education campaign for the general population, particularly aimed at young people.^52^

It was necessary, she continued, to influence young people's attitudes towards smoking and thus prevent them from taking it up. This focus on changing attitudes rather than using restrictive legislation endured in the years which followed. Schwarzhaupt explained that the BMG had negotiated with the VdC, resulting in the latter's commitment not to undertake advertising which was directed at young people.^53^ Within the Health Ministry, the starting point was negotiation with the tobacco industry to restrict advertising and education of the population, particularly young people, rather than legislation against either advertising or smoking itself.

This negotiation took the form of proposing a voluntary agreement with the implicit threat of legislation. In 1965, Secretary of State for Health, Bargatzky, noted that the BMG were continuing their efforts to negotiate a voluntary agreement on tobacco advertising. Answering a parliamentary question in 1965 about the increase in cigarette consumption in West Germany, despite media coverage of the US Surgeon General's report on smoking and health, Bargatzky took ownership of this proposed voluntary agreement, stating:

‘I have grounds to presume that the cigarette industry will shortly enter into an agreement, *suggested by me*, to restrict excessive advertising’ (author's emphasis).^54^

Bargatzky noted a draft bill to remove cigarette advertising from television, but stressed the need to await the voluntary agreement and its effect before taking any further steps.

The desire for self-regulation through voluntary agreements in the mid-1960s was a strategy adopted by the cigarette industry in countries such as the UK and the USA to avoid more restrictive legislation.^55^ Such agreements were inadequately enforced, and gave the perception of action on health with minimal disadvantage to the tobacco industry. The problems in West Germany related less to lack of enforcement (West German companies were keen to monitor infringements of the legislation amongst competitors, particularly US ones!) and more to the legality of such an agreement within the post-war context of decartelisation. In May 1964, BMG staff member, Dr Zoller, met with colleagues at the Federal Ministry of Economic Affairs (*Bundesministerium für Wirtschaft*, hereafter BMWi) to discuss the proposals. The memo of this meeting shows that the desire for a voluntary agreement met with opposition within the BMWi and was by no means fully endorsed even by all at the BMG. All present, including Zoller, shared the view that advertising was not a cause of youth smoking; excessive advertising was already covered by the *Nikotinverordnung*; there was no practical gain to be expected from a prohibition on tobacco advertising; and health education would have more of an effect. Nonetheless, Zoller stressed that Schwarzhaupt was determined to push ‘measures’ through, although he also explained that he had good ties to the cigarette industry funded Research Institute and that he had personally been able to prevent ‘misunderstandings’ arising.^56^ It is not clear what ‘misunderstandings’ are referred to; nonetheless the relationships between the BMWi, the cigarette industry and members of the BMG are clear. The final point which Zoller raised is crucial to understand the liberal approach to smoking in post-war West Germany. He questioned whether a voluntary agreement on cigarette advertising would infringe the 1957 Law Against Restraints on Competition (*Gesetz gegen Wettbewerbsbeschränkungen*, hereafter GWB).

Briefly put, the legitimacy of the post-war West German state was based upon economic freedom, within the social market economy, which was a reaction and response to previous decades of ‘economic authoritarianism’.^57^ As Berghahn has shown, Ludwig Erhard (the Minister of Finance from 1949 to 1963, and Chancellor of West Germany from 1963–66) was closely connected to, and influenced by, the arguments of the Freiburg School in the early 1940s. The Freiburg School opposed the planned economy of the Nazis, and argued for a post-war free market economy, which dismantled existing cartels, and was directed at civilian consumption.^58^ This distinctly German strand of liberal thought was known as Ordoliberalism after the journal published by the Freiburg School.^59^ Erhard himself set up an institute for research into consumerism, which was supported, among other industrialists, by Reemtsma.^60^ Later, in 1951, Erhard became a member of the internationally focused neo-liberal Mont Pelerin Society.^61^ In 1948, he had argued in a speech that ‘only a state that establishes both the freedom and the responsibility of its citizens can legitimately speak in the name of the people’.^62^ It is this interaction of freedom and responsibility which shaped West German smoking policy.

The US occupying authorities were also keen to prevent economic concentrations of power, and saw this as key to West Germany's integration into the new international community.^63^ While US planners advocated decartellisation, the US vision was slightly different from Ordoliberalism, as the US envisaged the survival of larger West German corporations which could compete on the global stage and drive European economic growth.^64^ This point explains why large companies like Reemtsma were allowed to rebuild after the war and were seen as important to West Germany's reconstruction.

In the early 1950s, the nascent neo-liberal climate countered any limits on competition. Long and protracted discussions to limit cartels and protect free competition led to the 1957 GWB which aimed to prevent monopolies and the distortion of the free market. For Erhard, the 1957 GWB was the economic equivalent to the 1949 *Grundgesetz* (Basic Law); both created the framework within which the liberal-democratic economy and society would operate.^65^ The 1957 GWB established the Federal Cartel Office (*Bundeskartellamt*), a body which would monitor the workings of the GWB. In practice, the GWB meant that the cigarette industry could not agree to limit advertising, or modify products in a certain way, without breaching this law, because of the perceived effects on free competition. Manufacturers had to show that such agreements were aimed at preventing harm. For example, car manufacturers had agreed not to advertise the top speed of their vehicles, and not to add radiator mascots which endangered others in traffic, in order to cut traffic accidents and fatalities.^66^

Thus, in order for the voluntary agreement on advertising not to breach the GWB, the cigarette industry had to be clear that it aimed to limit potential harm to the general public (a point which implied an acceptance of the health risks of smoking). In its original form, the voluntary agreement of 1965–66 reflected concern about smoking among young people, focusing on curtailing advertising seen to appeal to youth smokers, both in terms of the content and placing of advertising.^67^ The Federal Cartel Office objected to the agreement on the grounds that, in their view, such restrictions could not be seen as a health measure as the tobacco industry did not propose to restrict advertising expenditure as a whole. As far as the Federal Cartel Office were concerned, if the proposals would not lead to a reduction in cigarette industry profits (indicating a reduction in consumption), the agreement had no relevance in health terms, and therefore breached the terms of the law.^68^

Between 1964 and 1966, both the VdC and the BMG sought to convince the Federal Cartel Office that a voluntary agreement restricting advertising to young people would not affect competition in the cigarette market.^69^ Again, Bargatzky was clear that the initiative for the voluntary agreement had come from the BMG, and that its failure due to opposition from the Federal Cartel Office would threaten not only the credibility of the VdC, but that of the Federal Health Minister herself.^70^ In the end, the ‘qualitative’ sections of the agreement which limited the imagery used in advertising, such that it should not appeal to young people, were accepted but the ‘quantitative’ clause to limit the placing of print media and billboard advertising was not.^71^ Thus, the wider post-war economic commitment to free market competition expressed in law worked against restricting the tobacco trade in any meaningful way, and forced the cigarette industry and the Federal Health Ministry to cooperate to gain even minimal concessions.

## Teaching Responses to Advertising: Social and Political Influences

At the same time, the social climate of the late 1960s and early 1970s meant that a repressive approach towards smoking was politically unpalatable. The period between the late 1950s and the mid-1970s in West Germany was a period of transformation. The material factors ensuring a good standard of living for many West Germans were secured in the 1950s through the economic recovery and a robust social system.^72^ The 1960s saw rising disposable income and increased international travel changing people's outlook, while political and cultural alignment with the United States and other Western democracies set West Germany increasingly in a western way of life.^73^ A change of leadership from the conservative Christian Democrats to the Social Democrats, as well as a generational change in the social elite, provided the impetus to challenge conservative attitudes.

Liberalising protest movements were also an international phenomenon, spurred by opposition to the Vietnam War and nuclear weapons, and the demand for civil rights.^74^ In West Germany, examination of the National Socialist past and questions of democratic participation and plurality meant that such movements assumed heightened significance.^75^ Cultural transformation towards a more liberal state in the 1960s West Germany was, according to authors such as Ulrich Herbert, not only fast, but accompanied by an unusually high level of political and public acceptance.^76^ When Chancellor Willy Brandt entered office in 1969 with the slogan ‘Wir wollen mehr Demokratie wagen’, it was symbolic of the wider acceptance of these cultural changes.^77^

One aspect of this cultural shift was that anxieties about the negative effects of modern culture on young people were increasingly seen as outdated. Siegfried has shown that by the later 1960s young people were seen as the arbiters of modern modes of leisure, fashion and music, and that adults, while conflicted about some aspects of new lifestyles, sought to negotiate cultural change.^78^ The more open, liberal climate from the late 1960s meant that there was a need to maintain, and be seen to maintain, individual freedom as a fundamental condition of a democratic society. As theorists from Foucault onwards have shown in relation to the concept of ‘governmentality’, strategies of government in advanced liberal societies do not articulate traditional forms of authoritarian power, but gain validity, in the words of Nikolas Rose, through the ‘domains of market, civil society and citizenship’. The tasks of government are to foster self-regulation and self-control in order to govern ‘free citizens’.^79^ From 1967, health education campaigns at a federal level were designed and coordinated by the *Bundeszentrale für Gesundheitliche Aufklärung*, a body which was set up in place of the existing body, the *Deutsche Gesundheitsmuseum* (DGM).^80^ Frankenberg has called the BZGA ‘underfunded and inadequate’ and it is true that the BZGA suffered from funding constraints.^81^ Nonetheless, the campaign material produced by the BZGA was constantly cited by members of government as a key plank of the anti-smoking strategy, and provides an insight into the way in which various strategies of governmentality operated in relation to smoking. Smoking, alongside sex education, was one of the key campaign foci of the late 1960s; in the early 1970s, this broadened to include drugs education.^82^

From its inception in 1967, the BZGA adopted a strategy of evaluating and attempting to influence individual behaviour. The office took over work in progress from its predecessor, the DGM, including an anti-smoking brochure entitled *Was stimmt nun eigentlich?*.^83^ The BZGA also commissioned an extensive evaluation of smoking habits among young people, including gaining feedback on the brochure. Like studies in Britain at the time, this evaluation, was based on behavioural psychology.^84^ It was extensively referenced with international literature and was one of a series of such commissioned studies which drew heavily on English-language literature, demonstrating a use of expert knowledge beyond Germany as the basis of policy.^85^ There were also studies in West Germany looking at female smoking, similarly referenced, but male adult smoking culture remained beyond reach.^86^

The brochure, *Was stimmt nun eigentlich?*, explicitly addressed cigarette advertising, encouraging young people to think about the messages such advertising imparted. The timing of this brochure, a year after the voluntary agreement on cigarette advertising had been signed, suggests some acknowledgement that the agreement would achieve little. Archival material relating to the development of the brochure by the DGM initially shows that the cigarette industry was seen as a credible partner. This is distinct from the case in Britain where polarised discourses in relation to smoking and health were already beginning to emerge between the tobacco industry, on one hand, and medical and public health interests within and beyond government on the other.^87^ The content of the brochure came from both independent medical experts and scientific researchers at the industry-funded *Wissenschaftliche Forschungsstelle der Verband der Zigarettenindustrie*, including literature, statistical data, glossy images and previous advertising slogans.^88^ The BZGA do not appear to have continued links with the tobacco industry, suggesting that the creation of this office possibly had some effect in shifting the political alliances.

The evaluation of the brochure, conducted in 1968, suggested readers saw the content as an advertisement for the cigarette industry; interviewees were incredulous that it was intended to be against smoking.^89^ The evaluation discussed the reasons young people smoked: it was seen as a routine and accepted part of adult behaviour. Children observed that smoking was pleasurable for adults; that possessing a packet of cigarettes was seen to be important, and lacking cigarettes caused discomfort. Further, smoking was prevalent in popular culture: the cigarette was seen as the pre-requisite of modern heroes—the gangster, detective or secret agent of youth literature, for example. The young person wanted to participate in this positive image of the smoker, who was seen as ‘lively, modern, independent, chic, desirable, dynamic’, and ‘not too stiff or rigid in his principles’.^90^ The authors of the evaluation noted that young people identified with the smokers in the brochure, noting that, for young people, ‘the fear of not being up-to-date any longer [was] at least as strong, if not stronger, than the fear of not being physically fit any more’.^91^ Even the concept of fitness was inverted to make smoking a positive choice. Respondents suggested that smokers ‘could afford’ to smoke—a point understood in both financial and physical terms. Given that the dangers of smoking were well known by this point, being able to tolerate cigarettes was seen as a mark of a strong, solid man. Primarily, smoking was about being perceived as an adult.

On the other hand, non-smokers were seen as hen-pecked paragons of virtue, industrious people who were not only anxiously concerned about their own health, but wanted to educate and convert others to non-smoking. The report argued that ‘[n]on-smokers are not directly rejected, but often pitied, because they lack *joie de vivre* and pleasure. They are easily marked as outsiders’.^92^ If non-smoking was unattractive, regulation was seen as even more problematic. The evaluation concluded that prohibiting smoking would ‘just make it harder for young people to give up’ as smoking would become a means of protest.^93^ The report's authors were explicitly aware of the context of the generational conflicts of the late 1960s.

While British health education research focused on creating mass poster campaigns, the West German *Neue Trend* campaign was an attempt to engage with youth culture.^94^ It was multi-faceted, drawing in the popular youth magazine, *BRAVO*, creating mock fanzines and lesson plans, as well as using more traditional means such as posters. The media section of the campaign was run by a youth group called ‘Yeah Import from Great Britain Agents’ (Yeah for short; English in the original) in Munich with the support of the BZgA. This can be seen as an attempt to make the campaign hip and trendy, to distance the campaign from the government, and to draw on the trend towards greater internationalisation of young people.^95^ Material attempted to position smoking as no longer cool. Cartoons made the comparison between cigarettes and dummies, comparing smoking with a baby sucking a dummy, and undermining associations of smoking with masculine adulthood. The organisation also attempted to utilise peer pressure: readers were encouraged to set up non-smokers' clubs (called *Anti-Tabakschnuller Clubs* or Anti-Tobacco Dummy Clubs) and to send away for a free badge that read ‘I can smoke too’, allegedly making the need to prove this superfluous.^96^

Describing this campaign at the Second World Conference on Smoking and Health in 1971, Dr Fritsche, President of the BZGA, explained: ‘we found that young adolescent teenagers did not trust adults … to talk to them about smoking … material is only accepted when we use the channels that teenagers use themselves’. Fritsche was speaking alongside experts from countries worldwide. The delegate list shows that, while most were from British or North American backgrounds, West Germany was reasonably represented. Despite attempts to position the campaign as a teenage initiative, Fritsche's use of the word ‘we’ to describe the conceptualisation and implementation of the campaign shows where the impetus came from.^97^

The campaign was shaped by a recognition that leisure time in general, and teenage culture particularly, was changing, as were family lifestyles. Teenagers and young people had more free time, more disposable income and were growing up in a culture of affluence. This was also explicit in cigarette advertising: the introduction of a curriculum on alcohol and drug use, including smoking, explained to teachers that tobacco advertising promised a range of positive effects, including ‘Genuss im Stil der neuen Zeit (Progressivität)’ (Enjoyment in the style of the new era (progressivity)).^98^ This was the slogan from a successful advertising campaign of the early 1960s for Lord Extra cigarettes, showing that cigarette advertising captured broader desires to move on from the past. As Lord Extra was the first ‘light’ (low tar, low nicotine) cigarette introduced in West Germany (by the Hamburg-based branch of British American Tobacco), the slogan inferred that smoking in the post-war era addressed health concerns with a new style of smoking. The BZGA campaign indicated unease with rampant consumer culture, whilst recognising the attraction of new beginnings.

Health education centred on teaching children to develop a critical awareness of advertising, and presenting alternatives to smoking. One curriculum plan, aimed at 9–10 year olds, was made up of four two-hour sessions. The focus was on analysing images in cigarette advertising (motifs of friendship, leisure, happiness, youth, beauty, health). Children were encouraged to discuss whether these situations reflected the reality of when people smoked (under stress, in everyday life situations such as on a train, after eating, etc.). Pupils were asked to create a collage from magazines to show how they imagined non-smokers to be, differentiating them from the previous set of adverts discussed. Pupils were supposed to understand that there are no noticeable differences between smokers and non-smokers and be encouraged to think of alternatives to smoking—swimming, sport, cycling, doing art and crafts, and so on.^99^ This lesson did not mention health risks, focusing on patterns of behaviour and cultural understandings of smoking. The aim was to create positive values around non-smoking.

The next two lessons aimed to enable young people to be informed, rational and above all, confident citizens making responsible choices. These classes addressed peer pressure, using a strip cartoon of four friends, as research showed that friendship groups were a key site for smoking initiation. The lessons encouraged pupils to understand that conflict situations could develop within a group and to learn techniques to resolve such situations. The main character, Peter, met with his friends to go sledging; one had brought cigarettes and encouraged the others to try them instead of going sledging. Pupils were asked to consider Peter's possible reasons for smoking and for not smoking, and to enact these in a role play.^100^ In the next class, pupils were given two printed alternative endings to the story to consider. The endings, neither of which show Peter smoking, demonstrate, respectively, Peter ‘exercising his own free will’ and leaving, and ‘looking for support from another group member’. Both examples aimed to provide pupils with the means of taking responsibility for their own behavioural decisions and not simply submitting to the will of the group. Children were asked to talk about their experiences of peer pressure, and to consider how this made them feel.^101^

The last lesson was a more traditional lesson about the dangerous substances in cigarettes and cigarette smoke, demonstrated through experimentation.^102^ These lessons were part of a wider curriculum on alcohol, self-medication, advertising and health. Alongside interventions to children, parents and educators were targeted with a brochure outlining 12 tips for preventing drug use amongst children. The tips included making sure drugs (alcohol, cigarettes, tablets) were not accessible to children, raising the self-confidence and self-esteem of children, and teaching them ways of resolving emotional difficulties. Other tips included not setting a bad example; avoiding tensions within the family; being interested in children's schoolwork or apprenticeship; being willing to lend an ear about problems; providing children with places to play and spend leisure time; and raising consciousness about drugs. This went far beyond telling parents that smoking, drinking or taking drugs damages health, and even how to talk to their children about smoking and other drug use. Although there was no explicit connection, this broad awareness of health within society can be read through the lens of the 1948 World Health Organisation definition of health: ‘health is a state of complete physical, mental and social well-being, and not merely the absence of disease or infirmity’.^103^ The brochure suggested ways parents could improve family life and children's upbringing to remove the need for mood enhancing substances. The rationale was that drug use was a result of social and familial problems. As the brochure noted, ‘discontent with social and political relationships [could] be a trigger for drug misuse’.^104^ The text suggested engaging young people in doing social good—for example, helping young disabled people or the aged, supporting Amnesty International, or even owning and caring for a pet. To quote ‘[s]uch voluntary work helps against the pains of the world’, and was supposed to make young people into better citizens.^105^

By the mid-1970s, health education initiatives against smoking were part of wider campaigns against drug use. This can be seen in the context of increasing concerns about illegal drug use and the growth of the hippie culture at an international level.^106^ Staff from the BZGA were involved in UNESCO initiatves: a conference in 1972 set out a clear approach to anti-drugs education, suggesting campaigns against drug use should be about increasing the opportunities for intellectual, emotional, psychological and physiological development of young people. Drugs education should, the conference recommended, be part of general education and the responsibility of the whole community; health education programmes should engage not just with individuals, but the ‘whole environmental system in which the individual lives’.^107^ This approach drew on drug education programmes from the United States, with the aim that ‘[t]hrough the development and consolidation of the values of the young person, these will be then be internalised and communicated in behaviour’.^108^

Thus, by the early 1970s, health education on drug use, including smoking, clearly focused on young peoples' sense of self. ‘Values education’ linked to the ideas behind the late 1960s *Neue Trend* health education programme which sought to win pupils over emotionally to the positive values of non-smoking. There was also a recognition, after the youth protest movements of the later 1960s, that teenagers and young people should play an important part of shaping a more liberal and democratic society. As Siegfried has argued, young people increasingly took on a ‘role as paradigms for the handling of consumer society's opportunities [as well as] advocating the expansion of democratic ways’.^109^ The BZGA campaigns sought to to teach children to question the messages they recieved from those in authority and through consumer culture; to question the place of smoking in the world they were growing up in; and to question the behaviour of their parents and peers who smoked. Children were then ideally in a position to make an informed decision (although this decision was expected to be not to smoke), justify and be confident about it. Parents and educators were asked to provide a harmonious, peaceful environment where children could express themselves, contribute and become valued citizens in their own right. This would, in the eyes of the BZGA, remove the need to smoke.

The youth smoking campaign was the most prominent measure undertaken by the BZGA in the early 1970s to address smoking. Other measures included research into smoking cessation programmes, and a campaign to persuade official government bodies to restrict smoking in meetings and common spaces. The underlying rationale was ‘persuasion, not force’, with citizens' behaviour to be shaped through education and rational thought, through knowledge of risk, rather than regulation. This fits within the neo-liberal context where, as Johanna Oksala has argued, individuals are expected to make ‘strategic choices between the most effective means, ways and instruments [and] to be able to calculate costs, risks and possible returns’.^110^ The focus was not only on smoking and health, but the broader individual and social gains to be made by not smoking.

## The Route to Restrictions on TV Advertising

The emphasis on self-regulation and choice continued to frame policy discussions about smoking and health in the run up to the passage of the revised Food Law in 1974. The inclusion of a clause on cigarette advertising in this law had been mooted for some time; the earliest suggestion I have found is in correspondence around the *Nikotinverordnung* in 1959.^111^ Correspondence in 1966 refers to the use of legislative measures to prohibit advertising but is not specific what measures are meant. The prohibition of TV and radio advertising in 1974 did provide statutory regulation on smoking, but was far less than the cigarette industry expected and medical campaigners advocated. An internal memo from British American Tobacco of a 1969 meeting in Hamburg with G. F. Todd, director of the British Tobacco Research Council, anticipated warning labels, maximum tar levels, and minimum filter efficiencies as well as advertising restrictions. Schlenker (chairman of the board of the West German research institute, also present) was clear that West German cigarette manufacturers were willing to give up TV advertising if pressure for legislation became too great.^112^

This memo emphasised good levels of cooperation between the West German cigarette industry and its British counterparts, which ‘worked reasonably well’. The memo referred to information exchanges between Germany and Denmark; and between Britain, Holland and Norway. A 1969 document from the National Clearing House for Smoking and Health in the United States comparing tobacco policies in 22 countries was circulated and discussed by the cigarette industry internationally and government ministries in West Germany.^113^ The importance attached to this document suggests West German eyes looked outwards in formulating their policy response. This viewpoint can also be found within West German government: the Ministry of Economic Affairs compared smoking policies, cigarette consumption and levels of cigarette advertising in various Western countries.^114^ The cigarette industry also adopted a strategy of international comparison, referencing the lack of competition in state tobacco monopolies and arguing that an inability to advertise impacted on innovation in lower tar and lower nicotine brands.^115^

The revision of the Food Law began to be planned in 1965 but did not include anything relating to tobacco.^116^ Incorporating a clause on smoking was discussed in the Bundestag and the Bundesrat in late 1968; and initially was further ranging than restricting TV advertising—including prohibiting all additives to tobacco which would have had far-reaching implications for the import of US tobacco.^117^ By September 1969, correspondence between government and internal cigarette industry shows the BMJFG (*Bundesministerium für Jugend, Familie und Gesundheit*, which the BGM became in 1969) in talks with the VdC about advertising in particular. The 1966 voluntary agreement was thereaftfer extended to cover staggered withdrawal from TV and radio advertising. The agreement now included US multinational Philip Morris.^118^ The government supported the extension of the voluntary agreement on the grounds that ‘free co-operation’ would be more effective than going through legal channels.^119^ By 1973, a problem with the extended agreement was compliance by ‘outside’ (i.e. non West-German) companies. There were differences in approach between German firms who had a long standing history with the (West) German government and US multinational Philip Morris, whose undisclosed aim was to reduce the restraints which the advertising code placed on them.^120^

As in 1966, the *Bundeskartellamt* complained about the extended voluntary agreement being in breach of the GWB and advocated legislation to achieve the health aims.^121^ The office argued that agreements to restrict competition could only come into consideration when there were no legal means available, or such means were not timely. When such restriction was necessary, the Office argued, the state was better able to protect the interests of all interested parties.^122^ Quantitative agreements on restricting advertising, such as stopping TV and radio advertising, were against the law; but the agreement was authorised under paragraph 8 of the GWB, which allowed for Ministerial Authorisation.

Through the early 1970s, there was increased media and medical pressure to use the Food Law reform as a vehicle to enact legal restrictions on smoking, under the rubric of consumer protection.^123^ The cigarette industry held a consistent position as evidenced in the formal statement by the VdC to the Committee considering the Food Law in the Bundestag in March 1972, and in draft statements and correspondence. The VdC sought to present manufacturers as responsive to the health risks (highlighting industry research into the chemical components of tobacco smoke combined with animal experiments from the early 1950s onwards) and as responsible (having voluntarily restricted advertising). The cigarette industry also sought to minimise the dangers of smoking in a manner different to the denialism Proctor posits for the US case and earlier decades in Germany. By the early 1970s, there was an expressed acceptance of the health risks, but concerns were downplayed. The VdC distanced the West German product and therefore West German cigarette manufacturers from international health concerns in both time and space. The cigarette in West Germany in the early 1970s could not, they asserted, be compared with the cigarette for sale 30–40 years previously in the USA or the UK ‘either in respect of the quantity of smoke constituents or of its biological activity’.^124^

The VdC argued that advertising allowed the smoking public to make informed choices and promoted brand switching to ‘healthier’ alternatives (as the filter cigarette was constructed). The billions of Deutschmarks spent on advertising were justified in terms of public health, drawing on the rhetoric of choice. In a paragraph worth quoting in full to show how the debate was re-framed, the statement compared the risks of smoking to the social risks of illicit drugs:

If one accepts the thesis that human nature will not change in the forseeable future, then one can predict with certainty that humanity will employ pharmaceutical aids in its search for contentment. [It is] in the best interest of public health that this should be done with substances which involve the least risk both for the individual and for society as a whole. If one applies this principle to tobacco, then it must once again be emphasised that the danger*, serious though it may be*, exists in relation to the individual, as opposed to which the uncontrolled use of stronger, pharmacological substances throws up a multiplicty of social problems which today constitute a matter of great concern for the government [author's emphasis].^125^

The statement can be understood in the context of broader social concerns around recreational drug use, particularly among young people, alluded to at the opening of this paper. Whereas the BZGA sought to associate all drugs with social and personal harm, the cigarette industry sought to delineate between them, positioning the cigarette as the socially safer alternative. The industry position emphasised individual risk, which put smoking beyond the bounds of legitimate government action. The statement also paraphrased the liberal maxim: ‘(only) as much government as necessary; as much freedom as possible’, which has clear resonance with the economic liberalism developing from the 1950s.

The BMJFG, for their part, published a number of lengthy and hard-hitting parliamentary responses dealing fully with the international epidemiological and medical evidence against smoking. One in 1974 accepted the international consensus that action was needed, and rejected many of the tobacco industry arguments against such action. Nonetheless, ministers fell back on the rhetoric of informed choice, stating: ‘No-one intends to treat mature citizens as children and force healthy behaviour by laws or pressure. It must be the goal to awaken reason and criticism.’^126^

In short, despite being aware of and accepting the incontrovertible evidence against smoking on health grounds, the government accepted the premise that the state should not legislate on individual behaviours. Many such statements concluded by evoking the duality of freedom and responsibility. This position is in line with social and economic developments over the previous two decades, and can be understood in the context of the neo-liberal citizen exercising rational, self-interested choice. This context helps to explain the lack of action in the decades to follow.

## Conclusion

The development of smoking policy in the 1960s and early 1970s in West Germany shows that attitudes towards smoking were formed and maintained in spite of knowledge of the international epidemiological case against smoking, rather than in ignorance or denial of it. Debates around cigarette advertising within West Germany are part of the wider global story about an emerging response to smoking as a threat to health. Both the Federal Ministry of Health and the Federal Ministry of Economic Affairs were aware of and referred to the international context when discussing cigarette advertising. The health education response can also be seen within an international framework—not only because the impetus to action came from influential British and US government reports on smoking, but because the use of social psychology to inform policy was an approach that was also apparent elsewhere. By the 1970s, one sees the influence of values education in anti-drugs education, an approach specifically promoted by UNESCO. In West German health education in the 1970s, drugs were understood in the widest possible sense. Thus, the West German response to smoking was in many ways an international story, demonstrating a shift towards an international outlook.

Nonetheless, the particular national context of West Germany, particularly the experience of fascism and the economic and social rebuilding of the immediate post-war years, had an effect going beyond privacy regarding health matters. The neo-liberal economic context of post-war West Germany worked against any significant restriction of tobacco advertising or sales. The commitment to free trade within the social market economy was seen as integral to the maintenance of democracy in the post-fascist state. Health education and the research informing it can be located in the post-war move towards an advanced liberal society. The background of 1968 and the perceived generational conflict of the period prompted a desire to move away from a didactic form of health education which ‘talked down’ to children and young people. This was replaced by a form of health education which sought to create citizens of the future, capable of exercising a choice within a democratic society, rather than coercing them or frightening them into patterns of behaviour. This fits into analyses which stress neo-liberal forms of governmentality in shaping personal behaviour, in contrast to the overtly authoritarian anti-smoking campaigns of the Third Reich. The West German campaigns also contrast to the ‘shock’ and stigmatising tactics used in mass media advertising in the 1970s in the British case, and the earlier 1960s in West Germany.

The influence of the cigarette industry is undeniable throughout the period; roots of later twentieth-century relationships between the cigarette industry, scientists and the West German government can already be found in the immediate post-war years. Transnational networks were also emerging between West Germany, other European and US cigarette manufacturers. However, the cigarette industry in the 1950s was qualitatively and quantitatively different from that of the later twentieth century. The key players in the 1950s and 1960s in West Germany were West German family firm, Reemtsma, and the cigarette manufacturer's association, the VdC, which mainly included West German manufacturers. By the late 1970s, multinationals such as Philip Morris and RJ Reynolds were increasingly directing the agenda. By the 1980s, the West German government was negotiating with, and being lobbied, by a multinational, multibillion dollar industry which was not the case in the 1950s.

Finally, understanding West German tobacco policy in its international context provides an intriguing perspective on global issues around smoking and health. The early emphasis on individual freedom coupled with responsibility helps to explain why tobacco industry arguments fell on fertile ground in West Germany. The larger argument was not about smoking and health but about the appropriate role of the state, relating to both the legitimacy of market regulation and the regulation of personal behaviour. Just as links can be seen between the cigarette manufacturer, Reemstma, and the development of Ordoliberalism, British American Tobacco financially supported the British Institute of Economic Affairs, a right-wing think tank which disseminated neo-liberal ideals in Britain in the 1970s.^127^ Following these leads may help to explain why debates around smoking and health developed in similar ways in different Western countries in the 1960s and 1970s and why liberal arguments persisted in the face of increasing evidence of the health risks. There was not only an internationalisation of medical understandings of smoking as a risk to health, but the growth of transnational networks promoting discourses of individual economic and social freedom and challenging the role of the state in regulating individual behaviour, which went beyond the cigarette industry. The story of smoking in West Germany is an important part of these developments.
